# The large-sample asymptotic behaviour of quartet-based summary methods for species tree inference

**DOI:** 10.1007/s00285-022-01786-4

**Published:** 2022-08-17

**Authors:** Yao-ban Chan, Qiuyi Li, Celine Scornavacca

**Affiliations:** 1 School of Mathematics and Statistics / Melbourne Integrative Genomics, The University of Melbourne, Melbourne, 3010 VIC Australia; 2 Institut des Sciences de l’Evolution, Université Montpellier, CNRS, EPHE, IRD, Montpellier, 34095 France

**Keywords:** Species tree, Asymptotic behaviour, Sample complexity, Multispecies coalescent, 41A60, 60J90, 62E20, 92D15

## Abstract

Summary methods seek to infer a species tree from a set of gene trees. A desirable property of such methods is that of statistical consistency; that is, the probability of inferring the wrong species tree (the error probability) tends to 0 as the number of input gene trees becomes large. A popular paradigm is to infer a species tree that agrees with the maximum number of quartets from the input set of gene trees; this has been proved to be statistically consistent under several models of gene evolution. In this paper, we study the asymptotic behaviour of the error probability of such methods in this limit, and show that it decays exponentially. For a 4-taxon species tree, we derive a closed form for the asymptotic behaviour in terms of the probability that the gene evolution process produces the correct topology. We also derive bounds for the sample complexity (the number of gene trees required to infer the true species tree with a given probability), which outperform existing bounds. We then extend our results to bounds for the asymptotic behaviour of the error probability for any species tree, and compare these to the true error probability for some model species trees using simulations.

## Introduction

A central problem of phylogenetics is the inference of the evolutionary history of a group of species in the form of a species tree, where leaves represent extant species and internal vertices represent speciation events. Likewise, the gene families contained within the genomes of the species have their own evolutionary histories, which can also be represented by trees that can be considered as evolving ‘within’ the species tree. This implies that the gene trees are heavily dependent on the species trees.

Historically, species trees have been inferred using so-called ‘concatenation’ methods, where the sequences of individual genes are concatenated together to form a set of ‘species sequences’, which are then used as input to a tree-building method of choice, often maximum-likelihood based (e.g., RAxML, Stamatakis 2014). However, this carries the implicit assumption that the individual gene trees are identical to the species tree. This is not necessarily the case, due to a number of processes which cause discordance between the gene and species trees.

One such process is incomplete lineage sorting (Maddison 1997), which occurs when gene lineages do not coalesce immediately (going backwards in time) within a single species. This can cause branch length differences between the gene and species trees, and if alleles are maintained over a sequence of rapid speciations, even topological differences. Thus in recent years there has been a rapid growth of ‘summary’ methods for species tree inference, where gene families are identified and a gene tree built from the sequences of each individual family. The set of gene trees are then summarised in some way to produce a species tree. This paradigm acknowledges and incorporates the fact that gene trees may differ from each other and the species tree.

A popular summary method is ASTRAL (Mirarab et al. 2014; Mirarab and Warnow 2015), which works by solving the ‘maximum quartet support species tree’ (MQSST) problem. Each gene tree is decomposed into a number of gene quartets, with topologies determined by the gene tree. ASTRAL then uses a dynamic programming algorithm to find the species tree which maximises the number of gene quartets that agree with it. As this problem is NP-hard (Lafond and Scornavacca 2019), ASTRAL has two running modes: one which solves this problem exactly, and a faster heuristic which restricts the search space but does not guarantee optimality. We consider only the first mode in this paper.

The dominant statistical model for incomplete lineage sorting is the multispecies coalescent (MSC, Rannala and Yang 2003), which considers each species branch as a separate population in which Kingman’s coalescent (Kingman 1982) is run. The lineages at the top of each branch (going backwards in time) then become input to the coalescent at the bottom of its parent branch. This model provides exact probabilities of each gene tree (and gene tree topology) given a specified species tree. It has been shown for both unpartitioned and partitioned methods (Roch and Steel 2015; Roch et al. 2019) that under this model, there are species trees where concatenation methods are not *statistically consistent*: they do not reconstruct the true species tree with probability approaching 1 as the number of input gene trees becomes arbitrarily large. Conversely, ASTRAL is statistically consistent (for all species trees) under the multispecies coalescent (Mirarab et al. 2014), when input gene trees are considered to be correctly reconstructed from sequences.

In addition, it is well known that incomplete lineage sorting is not the only cause of discordance between gene and species trees. Discordance can also arise due to other genetic evolutionary processes such as gene duplication, gene loss, and lateral genetic transfer. Models of gene evolution incorporating duplication and loss only (DL) are well-established, dating back to Goodman (Goodman et al. 1979), as are models with duplication, loss, and transfer (DTL; see Doyon et al. 2011, for a review). These generally consider duplications and losses (and sometimes transfers) to occur as independent linear birth-death processes within each species branch. More recently, some unified models (DLCoal, Rasmussen and Kellis 2012, and MLMSC, Li et al. 2021) have been developed that account for both DTL and the population-level processes that cause incomplete lineage sorting.

For each of these models, it is of interest to determine if ASTRAL (and other summary methods) is statistically consistent. This becomes progressively more difficult as the models increase in complexity, but it has been proven that this is indeed the case for the DL model (Legried et al. 2021) and the DLCoal model (Markin and Eulenstein 2020). A numerical study (Yan et al. 2021) also looked at the practical accuracy of ASTRAL under the DLCoal model. The consistency of ASTRAL under the MLMSC (or, indeed, any models with transfers) remains unknown.

We approach this problem in a general way by not restricting our consideration to the MSC or any other particular model of gene evolution. Instead, we consider the asymptotic behaviour of the probability of inferring the wrong species tree (the *error probability*) as the number of input gene trees, *N*, becomes large. Rather surprisingly, while there has been considerable interest on whether the error probability goes to 0 or not (i.e., statistical consistency), no-one (to the best of our knowledge) has studied the asymptotic behaviour of the error probability when it does go to 0. Our results below show that the decay is exponential in nature, which is unsurprising but does not seem to have been theoretically proven before.

A very closely related work is that by Shekhar et al. (2017), who studied the *sample complexity* (the number of gene trees required to infer the true species tree with a given probability) of ASTRAL. They derived bounds for this quantity under the multispecies coalescent, and recently Hill et al. (2020) derived corresponding results under the DLCoal model. The focus of these papers is on the behaviour of the sample complexity as the length of the shortest branch of the species tree goes to 0, for a fixed (but small) error probability. Conversely, our results study the asymptotic behaviour of the error probability as the number of input gene trees becomes large, for a fixed species tree. However, their bounds are comparable in some situations to our results.

In this paper, we study the asymptotic behaviour of the error probability as $N \rightarrow \infty$. We first study the case of a 4-taxon species tree, and derive a closed form for the asymptotic behaviour in terms of the probability that the gene evolution process produces the correct topology. We also provide bounds for the error probability that hold for all *N*. This enables us to numerically derive corresponding bounds on the sample complexity for ASTRAL under the MSC, which outperform the bounds from Shekhar et al. (2017) in practice. We then extend our results to species trees with arbitrary numbers of taxa, resulting in bounds (both asymptotic and for all *N*) for the error probability for any species tree. Using simulations, we compare our bounds with the true error probability for some sample topologies.

## 4-taxon species tree

We first study the case where the species tree *S* has 4 taxa. We suppose that *N* unrooted gene trees are simulated from the species tree under an unspecified gene evolutionary process that has probability $p > \frac{1}{3}$ to produce the correct topology *S*, and equal probability $\frac{1-p}{2}$ to produce either of the other two possible topologies. (Although it is possible that the incorrect topologies may be produced with unequal probability, many models, such as the MSC, will produce them with equal probability.) These trees are then used as input to a quartet-based summary method to infer a species tree. Although we refer to this method as ASTRAL throughout this paper, we only require that it solves the MQSST problem. (In the case where the input gene trees have an equal maximum number of two or more different topologies, we assume that the method randomly chooses one to infer as the species tree.) Since $p > \frac{1}{3}$, we know that this method is statistically consistent.

We begin by defining a function that we will use repeatedly throughout this paper.

### Definition 1

Let *f*(*p*) be the function

$$\begin{aligned} f(p) = - 2c \ln c - (1-2c) \ln (1-2c) + c \ln p + (1-c) \ln \frac{1-p}{2},\end{aligned}$$

where

1 $$\begin{aligned} c = \frac{1}{2 + \sqrt{\frac{1-p}{2p}}}. \end{aligned}$$

**Fig. 1:**
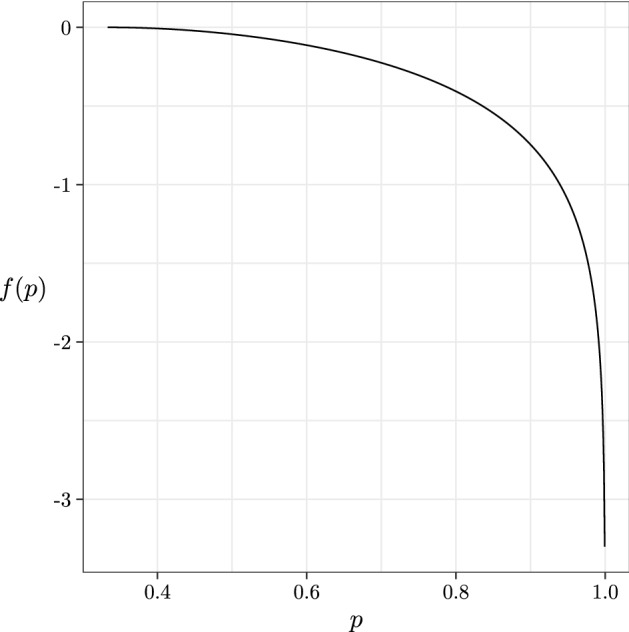
The function *f*(*p*)

We illustrate the behaviour of this function in Fig. 1. Important aspects to note are that it is defined (for our purposes) on the domain $p \in (\frac{1}{3},1)$, is always negative on this domain, and monotonically decreases to an asymptote at $p = 1$.

We now derive the central result of this paper: we show that the asymptotic behaviour of the error probability goes to 0 exponentially with *N*, and derive a closed form for the exponential constant that only depends on the probability *p* of producing the correct topology.

### Theorem 1

Given a 4-taxon species tree, let $p > \frac{1}{3}$ be the probability that the gene evolution process produces the correct topology. As the number of gene trees $N \rightarrow \infty$, the probability of inferring the wrong species tree with ASTRAL behaves as

$$\begin{aligned} \ln P(\text {error})\sim f(p) N. \end{aligned}$$

### Proof

Let $N_1, N_2, N_3$ be the number of gene trees produced with the three topologies, with $N_1$ being the correct one. (Obviously, $N_1+N_2+N_3 = N$.) Then $(N_1,N_2,N_3)$ has a multinomial distribution with parameters *N* and $(p, \frac{1-p}{2}, \frac{1-p}{2})$.

The correct species tree will be inferred if $N_1 > N_2, N_3$. Consider now the case $N_1 = N_2 = cN, N_3 = (1-2c)N$ for some $c \in [\frac{1}{3},1]$. In this case, the wrong species tree will be inferred with probability $\frac{1}{2}$. The probability of this event is:

$$\begin{aligned} P(N_1&= N_2 = cN, N_3= (1-2c)N)\\&= \frac{N!}{(cN)!(cN)!( (1-2c)N)!} p^{cN} \left( \frac{1-p}{2} \right) ^{cN} \left( \frac{1-p}{2} \right) ^{(1-2c)N} \\&= \frac{N!}{\left( (cN)!\right) ^2( (1-2c)N)!} p^{cN} \left( \frac{1-p}{2} \right) ^{(1-c)N}. \end{aligned}$$

(For brevity, we leave the $N_3 = (1-2c)N$ out in the following, as it is implied by $N_1 = N_2 = cN$.)

Using Stirling’s formula $\ln x! \sim x \ln x - x$,

$$\begin{aligned}&\ln P(N_1 = N_2 = cN) \\&\quad \sim N \ln N - N - 2(cN \ln cN - cN) - (1-2c)N \ln (1-2c)N \\&\qquad + (1-2c)N + cN \ln p + (1-c)N \ln \frac{1-p}{2}\\&\quad = \left[ - 2c \ln c - (1-2c) \ln (1-2c) + c \ln p + (1-c) \ln \frac{1-p}{2} \right] N. \end{aligned}$$

We now choose *c* to maximise the (log-)probability of this event:

$$\begin{aligned} \frac{d}{dc} \ln P(N_1 = N_2 = cN)&\sim \left[ - 2 \ln c - 2 + 2 \ln (1-2c) + 2 + \ln p -\ln \frac{1-p}{2}\right] N \\&= \left[ 2 \ln \frac{1-2c}{c} + \ln \frac{2p}{1-p} \right] N. \end{aligned}$$

Setting this to 0 yields

$$\begin{aligned} \ln \left( \frac{1}{c} - 2\right) ^2&= \ln \frac{1-p}{2p} \\ c&= \frac{1}{2 + \sqrt{\frac{1-p}{2p}}}. \end{aligned}$$

Note that there is no guarantee that *cN* is an integer for this value of *c*; however, as $N \rightarrow \infty$, it will become arbitrarily close to such a value.

We now claim that the probability of inferring the wrong species tree has the same asymptotic behaviour as $P(N_1 = N_2 = cN)$. Since this event will produce a wrong species tree with probability $\frac{1}{2}$, we have

$$\begin{aligned} P(\text {error})&\ge \frac{1}{2} P(N_1 = N_2 = cN) \\ \ln P(\text {error})&\ge \ln P(N_1 = N_2 = cN) - \ln 2. \end{aligned}$$

To show the opposite bound, consider that there are $\sim \frac{N^2}{2}$ possible combinations of $N_1, N_2, N_3$ which add to *N* (we can choose $N_1, N_2$ such that $N_1+N_2\le N$, and then $N_3$ is determined). Of these, $\frac{2}{3}$ produce the wrong species tree by symmetry. For each of these combinations $n_1, n_2, n_3$, we assume without loss of generality that $n_2 \ge n_1, n_3$. Then one of the following cases occurs:

- $n_1 = n_2$, so $P(N_1 = n_1, N_2 = n_2) \le P(N_1 = N_2 = cN)$ by construction of *c*;
- $$n_2 > n_1$$ and $$n_3 > n_1$$, so
  $$\begin{aligned} P(N_1 = n_1, N_2 = n_2)&= \frac{N!}{n_1!n_2!n_3!} p^{n_1} \left( \frac{1-p}{2}\right) ^{n_2+n_3} \\&< \frac{N!}{(n_1+1)!n_2!(n_3-1)!} p^{n_1+1} \left( \frac{1-p}{2}\right) ^{n_2+n_3-1} \\&= P(N_1 = n_1+1, N_2 = n_2). \end{aligned}$$
- $$n_2 > n_1 \ge n_3$$, so
  $$\begin{aligned} P(N_1 = n_1, N_2 = n_2)&= \frac{N!}{n_1!n_2!n_3!} p^{n_1} \left( \frac{1-p}{2}\right) ^{n_2+n_3} \\&\le \frac{N!}{n_1!(n_2-1)!(n_3+1)!} p^{n_1} \left( \frac{1-p}{2}\right) ^{n_2+n_3} \\&= P(N_1 = n_1, N_2 = n_2-1). \end{aligned}$$

We can recursively apply the latter two relations until $n_1 = n_2$, then use the first case. Thus each of these combinations has a probability which is bounded above by $P(N_1 = N_2 = cN)$.

Hence (with a slight abuse of notation, as the inequality only holds in the asymptotic limit)

$$\begin{aligned} P(\text {error})&\le \frac{N^2}{3} P(N_1 = N_2 = cN) \\ \ln P(\text {error})&\le \ln P(N_1 = N_2 = cN) + 2 \ln N - \ln 3. \end{aligned}$$

Since the two last terms are *o*(*N*), the claim is proved, as is the result. $\square$

**Note.** An initial attempt at deriving this result involved using a normal approximation to the multinomial; rather surprisingly (to us), this failed because the approximation of multinomial tail probabilities became inaccurate faster than the normal approximation became accurate.

This theorem leads to an immediate result on the Robinson-Foulds (RF) accuracy of the inferred species tree.

### Corollary 2

Under the conditions of Theorem 1, as the number of gene trees $N \rightarrow \infty$, the average RF distance between *S* and the tree inferred by ASTRAL behaves as

$$\begin{aligned} \ln (RF) \sim f(p) N.\end{aligned}$$

### Proof

This follows immediately from Theorem 1 and the relation $RF = 2P(\text {error})$.    $\square$

Theorem 1 can be extended to derive lower and upper bounds on the error probability, as we show in the following theorem. These bounds apply for all *N*, not just in the limiting case.

### Theorem 3

Under the conditions of Theorem 1, the probability of inferring the wrong species tree with ASTRAL is bounded below by

$$\begin{aligned} \ln P(\text {error})&\ge \left[ - 2c' \ln c' - (1-2c') \ln (1-2c') + c' \ln p + (1-c') \ln \frac{1-p}{2}\right] N \\&\quad - \ln N - \ln 4\pi c'(1-2c')^{1/2} - \frac{2-3c'}{12c'(1-2c')}\frac{1}{N} + \frac{1}{12N+1}, \end{aligned}$$

where $c'$ is the nearest number to

$$\begin{aligned} c = \frac{1}{2+\sqrt{\frac{1-p}{2p}}} \end{aligned}$$

such that $c'N$ is an integer. Likewise, it is bounded above by

$$\begin{aligned} \ln P(\text {error})&\le \max _c \left\{ \left[ - 2c \ln c - (1-2c) \ln (1-2c) + c \ln p + (1-c) \ln \frac{1-p}{2}\right] N \right. \\&\quad \left. + \ln \frac{N+1}{3} - \ln 2\pi c(1-2c)^{1/2} + \frac{1}{12N} - \frac{2}{12cN+1} + \frac{1}{12(1-2c)N+1} \right\} . \end{aligned}$$

The proof uses a bounded version of Stirling’s formula and is given in Appendix A.

Finally, we note that, depending on the gene evolution process, it is possible that the two incorrect topologies may not be generated with equal probability. This cannot happen for the MSC, but in models which include duplication it may occur for species trees that are not ultrametric, or if duplication rates are allowed to vary between branches. This can, and more easily, also happen in models including lateral gene transfers or introgression. In these cases, Theorem 1 can be generalised to the following result.

### Theorem 4

Given a 4-taxon species tree, let the probabilities of producing the three quartet topologies be $p_1 > p_2 \ge p_3$, where $p_1$ is the probability of producing the correct topology and $p_1 + p_2 + p_3 = 1$. As the number of gene trees $N \rightarrow \infty$, the probability of inferring the wrong species tree with ASTRAL behaves as

$$\begin{aligned}&\ln P(\text {error})\\&\quad \sim \Big [ -2c\ln c - (1-2c) \ln (1-2c) + c \ln p_1 p_2 + (1-2c) \ln (1-p_1-p_2)\Big ] N,\end{aligned}$$

where

$$\begin{aligned} c = \frac{1}{2 + \frac{1-p_1-p_2}{\sqrt{p_1p_2}}}.\end{aligned}$$

We sketch a proof in Appendix B, and also state a generalisation of Theorem 5 there. Note that, in models including lateral gene transfers or introgression, we can have extreme cases where the topology matching the species tree is not the most common one. In these cases, even our generalisations do not hold.

## General species tree

We now consider the case of a species tree *S* with an arbitrary number *n* of taxa. As before, we start by assuming that for any quartet, the gene evolution process produces the two incorrect topologies with equal probability.

To derive a lower bound on the asymptotic behaviour of the error probability, we require an assumption on the gene evolution process. Consider an internal branch *x* of the species tree which divides the extant species into four clades $A_x$, $B_x$, $C_x$, and $D_x$. Now consider two species quartets $(a_1,b_1,c_1,d_1)$ and $(a_2,b_2,c_2,d_2)$, such that one species is drawn from each clade for both quartets. We say that these two species quartets are *positively correlated* if the events of producing each of the three topologies are positively correlated between the two quartets; that is,

$$\begin{aligned}&P\big [ ((a_1,b_1), (c_1,d_1)) \cap ((a_2,b_2), (c_2,d_2)) \big ]\\&\quad \ge P\big [((a_1,b_1), (c_1,d_1))\big ]P\big [((a_2,b_2), (c_2,d_2))\big ],\end{aligned}$$

and likewise for the other two topologies. We then say that the gene evolution process is positively correlated if this property holds for all such pairs of quartets for all internal species branches. It is easy to see that the MSC is positively correlated, as there is a positive probability of the gene lineages from $a_1$ and $a_2$ coalescing before they reach the root, and likewise for the remaining three pairs of species.

### Theorem 5

Given a species tree *S*, assume the gene evolution process is positively correlated. Let $p_{\text {min}}$ be the minimum probability among all quartets in *S* of producing the correct topology. For any internal species branch $x \in E(S)$, let $A_x$, $B_x$, $C_x$, and $D_x$ be the four clades that it divides the leaves of *S* into, and let2Then the probability of inferring the wrong species tree with ASTRAL has the limiting behaviour (as $N \rightarrow \infty$)

$$\begin{aligned} \ln P(\text {error})\sim \alpha N,\end{aligned}$$

where the (logarithm of the) growth constant $\alpha$ is bounded by

3 $$\begin{aligned} \max _x f(p_x) |A_{x}||B_{x}||C_{x}||D_{x}| \le \alpha \le f(p_{\text {min}}).\end{aligned}$$

### Proof

To derive the upper bound, a sufficient (but not necessary) condition to infer the correct species tree is that each of the $$\left( {\begin{array}{c}n\\ 4\end{array}}\right) $$ species quartets have a plurality of gene quartets with the correct topology. (We use the word ‘plurality’ to mean that more gene quartets have the correct topology than any other, not that more than half the gene quartets have the correct topology.) Let $T_i$ be the event that species quartet *i* has a majority of gene quartets with the correct topology. Then

$$\begin{aligned} P(\text {error})&\le P\left( \cup _i T'_i\right) \\&\le \sum _i P(T'_i) \\&\le \left( {\begin{array}{c}n\\ 4\end{array}}\right) \max _i P(T'_i). \end{aligned}$$

Clearly, the probability of $T'_i$ is maximised for the species quartet that has the minimum probability $p_{\text {min}}$ of producing the correct topology (the smaller the probability of producing the correct topology, the smaller the probability that the majority of quartets have the correct topology). The upper bound then follows from Theorem 1.

To derive the lower bound, consider an internal species branch *x*, and consider the species quartets that contain one species from each of the clades $A_x, B_x, C_x, D_x$. There are $|A_{x}||B_{x}||C_{x}||D_{x}|$ such species quartets, which generate $N|A_{x}||B_{x}||C_{x}||D_{x}|$ gene quartets. A necessary (but not sufficient) condition to infer the correct species tree is that the majority of these gene quartets have the correct topology. To see this, suppose that the correct species tree is inferred, but the majority of these quartets have the wrong topology. In this case, performing a nearest-neighbour interchange move on *x* to this wrong topology will result in a species tree that agrees with more of these quartets, and not change the agreement with any other quartets. This improves the quartet score, a contradiction.

Let $E_x$ be the event that the majority of these gene quartets have the same wrong topology.

Now although gene quartets from different gene families are independent, this is not the case for gene quartets from the same gene family. Since the gene evolution process is positively correlated, the probability of $E_x$ is bounded below by the probability that the majority of the gene quartets have the same wrong topology if they were all generated independently. Thus we have

$$\begin{aligned} P(\text {error})&\ge P(E_x) \\&\ge P(E_x \mid \text {quartets generated independently}) \\&\ge P(E_x \mid \text {quartets generated independently with correct probability}\, p_x). \end{aligned}$$

We now apply the same argument as in Theorem 1, with the difference that instead of *N* gene quartets, we have $N|A_{x}||B_{x}||C_{x}||D_{x}|$ quartets. Since we can choose the *x* which maximises the resulting bound, this produces the lower bound.

Finally, because both the lower and upper bounds are *O*(*N*), we can say that the error probability also has this limiting behaviour as $N \rightarrow \infty$. $\square$

Note in particular that the upper bound does not depend on *n*, the size of the species tree, whereas the lower bound does.

While the derivation of the upper bound is very similar to that in Shekhar et al. (2017), the lower bound is qualitatively different. In that paper, they show the existence of a species tree for which the error probability is bounded below by a specified value. Our result is more general, in that it bounds the error for *any* species tree. (On the other hand, this means that their lower bound can be tighter than ours.)

Note also that under the MSC, the probability of producing the correct topology for a quartet depends only on the length of the internal branch; thus all species quartets (*a*, *b*, *c*, *d*) in (2) have exactly the same chance of producing the correct topology for a branch *x*, and there is no maximisation required to calculate $p_x$.

It is worth considering what sort of branch *x* will tend to maximise the lower bound. Since *f*(*p*) is a negative and decreasing function, it is maximised for small *p*. However, we also wish to simultaneously minimise $|A_{x}||B_{x}||C_{x}||D_{x}|$; this generally occurs when *x* is a branch near the leaves, so that three of the four clades are small. The smallest possible value for this quantity is either $n - 3$ (if *x* is the internal branch of a subtree ( (*a*, *b*), *c*)) or $2(n-2)$ (if no such subtree exists and *x* is one of the internal branches of a subtree ( (*a*, *b*), (*c*, *d*) )). Note that the first case in particular corresponds to the construction used to prove Claim 2.1 in Shekhar et al. (2017). Therefore, the branch that maximises the lower bound will usually be a short branch close to the tips of the species tree; this is the kind of branch that is most difficult to infer correctly.

Using similar arguments to Theorem 3, we can bound the error probability for general trees for all *N*; we state this result without proof in Appendix D.

## Sample complexity under the multispecies coalescent

The results in the previous sections apply under any model of gene evolution, requiring only the probability *p* of producing the correct topology. The most-studied model for this purpose is the multispecies coalescent, under which *p* depends only on the length of the internal branch *l* (in coalescent units), given by the relation $p = 1 - \frac{2}{3} e^{-l}$. (We change the notation here from Shekhar et al. 2017, where the length of the internal branch is denoted *f*, but we have already used this notation for the asymptotic growth constant function.) Substituting this relation into Theorem 1 gives the following result for the sample complexity of ASTRAL under the MSC on a 4-taxon species tree.

### Theorem 6

Under the multispecies coalescent, the minimum number of genes required to correctly reconstruct a 4-taxon species tree with ASTRAL with probability at least $1-\epsilon$ grows asymptotically (as $\epsilon \rightarrow 0$) as

$$\begin{aligned} N \sim g(l) \ln \epsilon ,\end{aligned}$$

where *g*(*l*) is the function

$$\begin{aligned} g(l) = \frac{1}{f(1-\frac{2}{3}e^{-l})}.\end{aligned}$$

As $$l \rightarrow 0$$,

$$\begin{aligned}g(l) \sim -\frac{4}{3} l^{-2}.\end{aligned}$$

### Proof

The first expression follows immediately from setting $P(error) = \epsilon$ in Theorem 1.

To derive the $l \rightarrow 0$ asymptotic behaviour, we expand (1) as a Maclaurin series for *c* in *l*:

$$\begin{aligned} c&= \frac{1}{2 + \sqrt{\frac{\frac{2}{3} e^{-l}}{2 - \frac{4}{3} e^{-l}}}} \\&= \frac{1}{3} + \frac{1}{6} l - \frac{5}{24} l^2 + O(l^3). \end{aligned}$$

Substituting this and $p = 1 - \frac{2}{3}e^{-l}$ into the expression for *f*(*p*) and expanding as a Maclaurin series in *l* gives the asymptotic behaviour for *g*(*l*). A more detailed derivation is given in Appendix C. $\square$

Corresponding bounds for the sample complexity are given in Shekhar et al. (2017). In the case $n = 4$, their bounds are (in our notation)

$$\begin{aligned} \left[ \frac{1}{2} \Phi ^{-1}\left( \frac{1}{4} + \frac{\epsilon }{2}\right) \right] ^2 l^{-2} \le N \le \frac{9}{2} \ln \left( \frac{4}{\epsilon }\right) \frac{1}{(1-e^{-l})^2},\end{aligned}$$

where $\Phi$ is the normal cdf. We see that the asymptotic behaviour of our expression agrees with the upper bound, as they are both $O(l^{-2} \ln \epsilon )$. The lower bound is not directly comparable, as it only applies for values of *l* below a threshold depending on $\epsilon$. Thus it is only suited to analysis of the case $l \rightarrow 0$ for fixed $\epsilon$, not $\epsilon \rightarrow 0$ for fixed *l*.

**Fig. 2:**
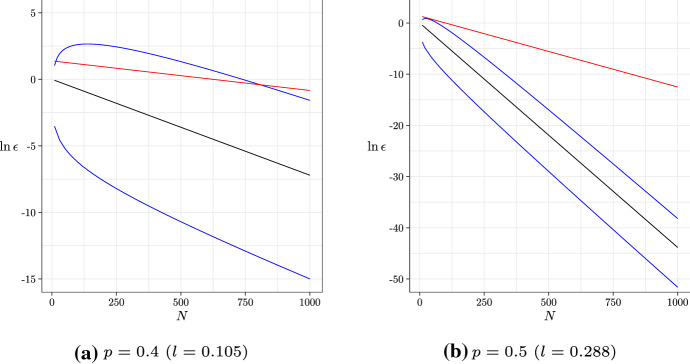
The logarithm of the error probability (for a 4-taxon species tree) vs *N*, for fixed *p* (fixed *l* under the MSC). We show the asymptotic behaviour (black), our bounds (blue), and the upper bound of Shekhar et al. (2017) (red) (color figure online)

We can derive bounds on the error probability for all *N* under the MSC from Theorem 3, by substituting the relation for *p*. In Fig. 2, we compare the asymptotic behaviour for the error probability against our bounds and the upper bound of Shekhar et al. (2017). We can see that our upper bound outperforms that of Shekhar et al. (2017) in the limit $N \rightarrow \infty$, and in general for large *l*; however theirs works better for small *N* and *l*. We can also see that the asymptotic behaviour lies between our bounds, which is expected in the asymptotic limit but appears to hold for all *N* anyway.

**Fig. 3:**
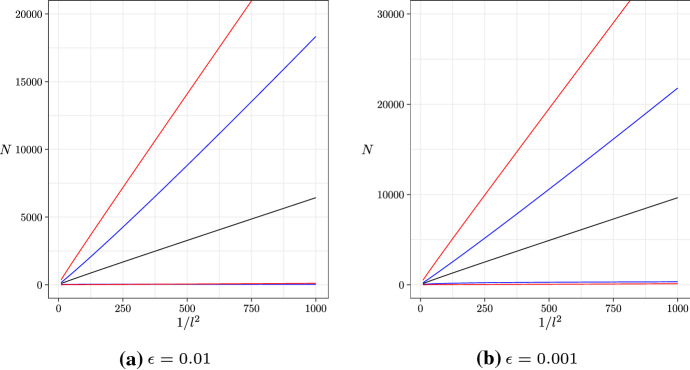
The sample complexity of ASTRAL (for a 4-taxon species tree) vs $1/l^2$, for fixed $\epsilon$. We show the asymptotic behaviour (black), our bounds (blue), and the bounds of Shekhar et al. (2017) (red) (color figure online)

We can also derive bounds for the sample complexity from Theorem 3 by solving for *N* numerically (an analytical inversion is likely impossible). We show the results of this in Fig. 3. We can see that our bounds, particularly the upper bound, outperform the bounds of Shekhar et al. (2017). This is because we require very large *N* to reach the desired error probability, so the better asymptotic properties of our bounds result in tighter bounds than that of Shekhar et al. (2017), which outperform ours for low *N* but have worse asymptotic properties.

Finally, we state bounds for the sample complexity under the MSC for the case of a species tree with an arbitrary number *n* of taxa.

### Theorem 7

Under the multispecies coalescent, the minimum number of genes required to correctly reconstruct a species tree with ASTRAL with probability at least $1-\epsilon$ grows asymptotically (as $\epsilon \rightarrow 0$) as

$$\begin{aligned}N \sim \frac{1}{\alpha }\ln \epsilon ,\end{aligned}$$

where

$$\begin{aligned} g(l_{\text {min}}) \le \frac{1}{\alpha }\le \frac{g(l_{x_{\text {max}}})}{|A_{x_{\text {max}}}||B_{x_{\text {max}}}||C_{x_{\text {max}}}||D_{x_{\text {max}}}|}, \end{aligned}$$

where $l_{min}$ is the length of the shortest internal species branch and $x_{\text {max}}$ is the branch that maximises the lower bound in (3).

## Simulations

**Fig. 4:**
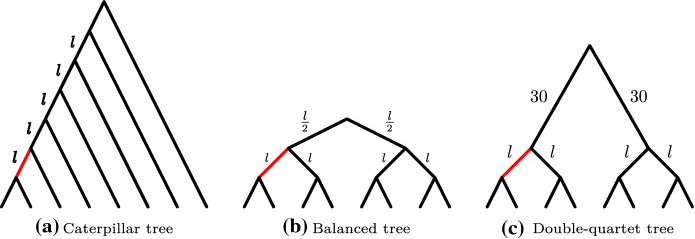
The three model trees. The branch that provides the lower bound is highlighted in red (color figure online)

We follow the simulations of Shekhar et al. (2017) and study three model species trees with $n = 8$ species, shown in Fig. 4. The caterpillar tree is maximally unbalanced, while the balanced tree is maximally balanced; both have all internal branches with length *l*. The ‘double-quartet’ tree has the same topology as the balanced tree, but with a very long internal branch (separating the two quartets) and all other branches of length *l*.

The branch that provides the best lower bound in Theorem 5 for each tree is highlighted in Fig. 4. Because all internal branches (save the long branch in the double-quartet tree) have the same length (and therefore the same probability of producing the correct topology under the MSC), this branch is the one that minimises $|A_{x}||B_{x}||C_{x}||D_{x}|$. The value of this quantity is 5, 8, and 8 for the caterpillar, balanced, and double-quartet trees respectively, which also gives the factors by which the lower bound is (negatively) greater than the upper bound. For these trees, the upper bounds are identical.

We simulate *N* independent gene trees under the MSC model for a range of *N* from 10 to 60000. The gene trees are then input into ASTRAL to infer a species tree, which is then compared with the true species tree. This is done for a range of *l* from 0.005 to 0.1 (with $l^{-2}$ evenly spaced as in Shekhar et al. 2017), with varying numbers of replicates (always at least 2000) for each value of *N* and *l*. We then fit a linear regression to $\ln \epsilon$ against *N* to calculate the growth constant $\alpha$.

**Fig. 5:**
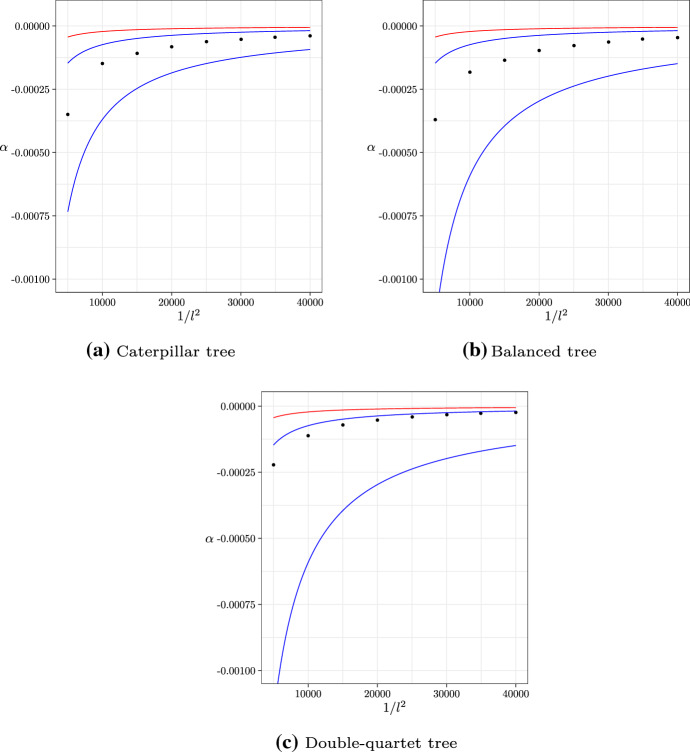
Growth constants for $\ln \epsilon$ for the three model trees, with our asymptotic bounds (blue) and the upper bound of Shekhar et al. (2017) (red) (color figure online)

In Fig. 5, we show the growth constants for the three trees against $l^{-2}$, together with our asymptotic bounds from Theorem 5 and the upper bound of Shekhar et al. (2017). (As mentioned above, the lower bound of Shekhar et al. 2017, cannot be compared here.) We can clearly see that our formulas do indeed bound the asymptotic growth constants, and moreover that we provide a tighter upper bound on the asymptotic behaviour than Shekhar et al. (2017).

In general, the true growth rate is closer to our upper bound than our lower bound. This is unsurprising considering that the lower bound is derived by considering all quartets corresponding to the same internal branch as independent, a large relaxation for the highly positively-correlated MSC. We also observe the pattern observed in Shekhar et al. (2017) that ASTRAL performs worse (the growth rate of the log-error probability is smaller negatively) on the double-quartet tree than the other two trees; see Shekhar et al. (2017) for a discussion of why this is so.

## Discussion

In this paper, we have derived the asymptotic behaviour of the error probability (the probability of inferring the wrong species tree) of ASTRAL in the large-sample limit (as the number of input gene trees becomes large). In particular, we show that the error probability goes to 0 exponentially with respect to *N*, and derive a closed form for the growth constant of this exponential behaviour exactly for the simplest case of a 4-taxon species tree. We calculate bounds on the growth constant for species trees with arbitrary number of taxa, and extend our results to rigorous bounds for any *N* for both the 4-taxon and general species tree. Our results improve previous bounds on the sample complexity from Shekhar et al. (2017).

It is true that in practice each species only has a finite number of genes (where a gene is simply a non-recombining locus in a genome); thus *N* has a natural boundary which it cannot go beyond. On the other hand, the increasing amount of whole-genome data available does allow a greater and greater number of genes (and therefore gene trees) to be used in species tree inference, and so an exploration of the behaviour of these methods in the large-*N* limit is of practical importance. In particular, our results are good enough to be useful in the question of determining the sample complexity, which is of great practical interest as it allows one to estimate *a priori* how many gene trees need to be extracted from the genome for use in species tree inference.

Our results are quite general, in the sense that although they are expressed in relation to ASTRAL, they apply for any species tree inference method which solves the maximum quartet support species tree problem. Nor are they limited to the multispecies coalescent model; although this is the dominant model at the moment, this may change with the recent rise of unified models that also include DTL processes, such as DLCoal or MLMSC. Our results (possibly apart from the lower bound for *n* species, which additionally requires positive correlation) apply equally well to these models, and to future models that may additionally include other processes. We require only the ability to calculate the probability of the gene evolution process generating a correct quartet topology. While this is not always an easy task theoretically under the more complex models, it can often be estimated very accurately via simulations on 4-taxon species trees (vastly more efficient than conducting simulations over many species).

One restriction on the gene evolution process that we require for our lower bound is that it must be positively correlated. This seems a natural requirement, and it is indeed easy to show that it holds for the MSC (as discussed above). However, it is not trivial to extend this argument to more complicated models such as DLCoal or MLMSC, and it remains an open question whether these models are indeed positively correlated or not.

Finally, we note that the important practical consideration of whether the gene trees are reconstructed with or without error does not affect our theoretical results in Sects. 2 and 3. We only require the probability *p* of producing the correct quartet topology (or in the more general case, the probabilities $p_1,p_2,p_3$ of the three topologies). If there is gene tree error, we can simply consider *p* as the probability of reconstructing, with error, the correct topology (likewise in the more general case). For our results to hold, it only matters that $p > \frac{1}{3}$, so that we are in the statistically consistent regime. As discussed in Shekhar et al. (2017), this will hold under a simple model where the gene tree error proportion is less than or equal to $\frac{2}{3}$, and errors are unbiased and independent between quartets.

For Sects. 4 and 5, the presence of gene tree error does affect the formula $p = 1 - \frac{2}{3} e^{-l}$, and so the asymptotic behaviour for the sample complexity becomes a lower bound in practice.

## Appendix A Proof of Theorem 3

We start by following the proof of Theorem 1. Let $N_1$, $N_2$, and $N_3$ be the number of gene trees produced with the three possible topologies as before. As before, we have

$$\begin{aligned} P(N_1 = N_2 = cN, N_3 = (1-2c)N) = \frac{N!}{\left( (cN)!\right) ^2( (1-2c)N)!} p^{cN} \left( \frac{1-p}{2} \right) ^{(1-c)N}.\end{aligned}$$

We first derive the lower bound by using a bounded version of Stirling’s formula (Robbins 1955),

$$\begin{aligned} x \ln x - x + \frac{1}{2} \ln 2 \pi x + \frac{1}{12 x+1} \le \ln x! \le x \ln x - x + \frac{1}{2} \ln 2 \pi x + \frac{1}{12 x}.\end{aligned}$$

This gives

$$\begin{aligned}&\ln P(N_1 = N_2 = cN) \\&\quad \ge N \ln N - N + \frac{1}{2} \ln 2 \pi N + \frac{1}{12N+1}\\&\qquad - 2\left( cN \ln cN - cN + \frac{1}{2} \ln 2 \pi cN + \frac{1}{12 cN}\right) \\&\qquad - (1-2c)N \ln (1-2c)N + (1-2c)N \\&\qquad - \frac{1}{2} \ln 2\pi (1-2c)N - \frac{1}{12(1-2c)N}\\&\qquad + cN \ln p + (1-c)N \ln \frac{1-p}{2}\\&= \left[ - 2c \ln c - (1-2c) \ln (1-2c) + c \ln p + (1-c) \ln \frac{1-p}{2}\right] N \\&\qquad - \ln N - \ln 2\pi c(1-2c)^{1/2} - \frac{2-3c}{12c(1-2c)N} + \frac{1}{12N+1}. \end{aligned}$$

Half of this probability gives a lower bound on the error probability for any *c*, as long as *cN* is an integer. We thus choose *c* as before to maximise *f*(*p*), but round it so that *cN* is an integer. (In theory, a marginally better bound could be found by optimising over possible values of *c*, but the integer constraint makes this difficult.)

We now derive the upper bound using the same bounds for Stirling’s formula. This gives

$$\begin{aligned}&\ln P(N_1 = N_2 = cN)\\&\quad \le N \ln N - N + \frac{1}{2} \ln 2 \pi N + \frac{1}{12N} \\&\qquad - 2\left( cN \ln cN - cN + \frac{1}{2} \ln 2 \pi cN + \frac{1}{12 cN+1}\right) \\&\qquad - (1-2c)N \ln (1-2c)N + (1-2c)N\\&\qquad - \frac{1}{2} \ln 2\pi (1-2c)N - \frac{1}{12(1-2c)N+1}\\&\qquad + cN \ln p + (1-c)N \ln \frac{1-p}{2}\\&\quad = \left[ - 2c \ln c - (1-2c) \ln (1-2c) + c \ln p + (1-c) \ln \frac{1-p}{2}\right] N \\&\qquad - \ln N - \ln 2\pi c(1-2c)^{1/2} + \frac{1}{12N} - \frac{2}{12 cN+1} + \frac{1}{12(1-2c)N+1}. \end{aligned}$$

We now choose *c* to maximise the probability of this event (we do not need to constrain *cN* to be an integer here). As shown in the proof of Theorem 1, this provides an upper bound for the probability of any specific combination of $N_1,N_2,N_3$ which adds to *N* and may produce the wrong species tree.

Now there are $\frac{N(N+1)}{2}$ possible combinations of $N_1,N_2,N_3$ which add to *N*. By symmetry, $\frac{2}{3}$ of these combinations produce the wrong species tree. Thus

$$\begin{aligned} \ln P(\text {error})\le \ln P(N_1 = N_2 = cN) + \ln \frac{N(N+1)}{3}.\end{aligned}$$

This gives the upper bound.

## Appendix B Non-equivalent alternative topologies

Here we extend our results to the case where the two incorrect topologies need not be generated with equal probability. We start by briefly sketching a proof of Theorem 4, which proceeds exactly as for Theorem 1 with small variations. Firstly, $(N_1,N_2,N_3)$ has a multinomial distribution with parameters *N* and $(p_1,p_2,p_3)$, which gives

$$\begin{aligned} P(N_1 = N_2 = cN, N_3 = (1-2c)N) = \frac{N!}{\left( (cN)!\right) ^2( (1-2c)N)!} p_1^{cN} p_2^{cN} p_3^{(1-2c)N}.\end{aligned}$$

Applying Stirling’s formula gives

$$\begin{aligned}&\ln P(N_1 = N_2 = cN) \\&\quad \sim \left[ - 2c \ln c - (1-2c) \ln (1-2c) + c \ln p_1 p_2 + (1-2c) \ln p_3 \right] N \\&\quad \frac{d}{dc} \ln P(N_1 = N_2 = cN) \sim \left[ 2 \ln \frac{1-2c}{c} + \ln \frac{p_1p_2}{p_3^2} \right] N. \end{aligned}$$

Setting to 0 and solving for *c* yields

$$\begin{aligned} c = \frac{1}{2 + \frac{p_3}{\sqrt{p_1p_2}}}.\end{aligned}$$

Substituting $p_3 = 1 - p_1 - p_2$ into these expressions gives the asymptotic expression for the error probability.

There is an extra difficulty with showing that each specific error term is bounded above by $P(N_1 = N_2 = cN)$, as the third relation $P(N_1=n_1,N_2=n_2) \le P(N_1=n_1,N_2=n_2-1)$ for $n_2 > n_1 \ge n_3$ used in the proof of Theorem 1 does not hold. We can instead use the relation $P(N_1=n_1,N_2=n_2) \le P(N_1=n_1+1,N_2=n_2-1)$ for $n_2 > n_1$, but this only shows that each error term is bounded above by either $P(N_1 = N_2 = cN)$ or $P(N_1 = cN, N_2 = cN+1)$. However, since these terms have the same asymptotic behaviour as $N \rightarrow \infty$, the result still holds.

We now state a generalisation for Theorem 5. The proof is sufficiently straightforward that we will omit it.

### Definition 2

Let $f(p_1,p_2)$ be the function

$$\begin{aligned} f(p_1,p_2) = - 2c \ln c - (1-2c) \ln (1-2c) + c \ln p_1p_2 + (1-2c) \ln (1-p_1-p_2),\end{aligned}$$

where

$$\begin{aligned} c = \frac{1}{2 + \frac{1-p_1-p_2}{\sqrt{p_1p_2}}}.\end{aligned}$$

### Theorem 8

Given a species tree *S*, assume the gene evolution process is positively correlated. For any species quartet (*a*, *b*, *c*, *d*), let the probabilities of producing the three quartet topologies be $p_1(a,b,c,d) > p_2(a,b,c,d) \ge p_3(a,b,c,d)$, where $p_1(a,b,c,d)$ is the probability of producing the correct topology and $p_1(a,b,c,d) + p_2(a,b,c,d) + p_3(a,b,c,d) = 1$. For any internal species branch $x \in E(S)$, let $A_x$, $B_x$, $C_x$, and $D_x$ be the four clades that it divides the leaves of *S* into.

Then the probability of inferring the wrong species tree with ASTRAL has the limiting behaviour (as $N \rightarrow \infty$)

$$\begin{aligned} \ln P(\text {error})\sim \alpha N, \end{aligned}$$

where the (logarithm of the) growth constant $\alpha$ is bounded by

$$\begin{aligned} \alpha&\ge \max _x \min _{a \in A_x,b\in B_x,c\in C_x,d\in D_x} f(p_1(a,b,c,d),p_2(a,b,c,d)) |A_{x}||B_{x}||C_{x}||D_{x}|, \\ \alpha&\le \max _{(a,b,c,d)} f(p_1(a,b,c,d),p_2(a,b,c,d)). \end{aligned}$$

## Appendix C Expanded proof of Theorem 6

We firstly expand the Maclaurin series for *c* and *p*:

$$\begin{aligned} c&= \frac{1}{2 + \sqrt{\frac{\frac{2}{3} e^{-l}}{2 - \frac{4}{3} e^{-l}}}} \\&= \frac{1}{3} + \frac{1}{6} l - \frac{5}{24} l^2 + O(l^3) \\ p&= 1 - \frac{2}{3} e^{-l} \\&= \frac{1}{3} + \frac{2}{3} l - \frac{1}{3} l^2 + O(l^2). \end{aligned}$$

Then,

$$\begin{aligned} -2c \ln c&= \frac{2}{3} \ln 3 + \left( \frac{1}{3} \ln 3 - \frac{1}{3}\right) l + \left( - \frac{5}{12} \ln 3 + \frac{1}{3} \right) l^2 + O(l^3) \\ -(1-2c)\ln (1-2c)&= \frac{1}{3} \ln 3 + \left( - \frac{1}{3} \ln 3 + \frac{1}{3} \right) l + \left( \frac{5}{12} \ln 3 - \frac{7}{12}\right) l^2 + O(l^3) \\ c \ln p&= -\frac{1}{3} \ln 3 + \left( -\frac{1}{6} \ln 3 + \frac{2}{3}\right) l + \left( \frac{5}{24} \ln 3 - \frac{2}{3} \right) l^2 + O(l^3) \\ (1-c)\ln \frac{1-p}{2}&= -\frac{2}{3} \ln 3 + \left( \frac{1}{6} \ln 3 - \frac{2}{3}\right) l + \left( - \frac{5}{24} \ln 3 + \frac{1}{6} \right) l^2 + O(l^3). \end{aligned}$$

Therefore,

$$\begin{aligned} f(p) = -\frac{3}{4} l^2 + O(l^3).\end{aligned}$$

Series expansions were performed with the aid of WolframAlpha.

## Appendix D Bounds for general species trees for all *N*

### Theorem 9

Given a species tree *S*, let $p_{\text {min}}$ be defined as in Theorem 5, and

$$\begin{aligned} x_{\text {max}}&= {\mathop {{{\,\mathrm{arg\,max}\,}}}\limits _{x}} f(p_x) |A_{x}||B_{x}||C_{x}||D_{x}|, \\ p_{\text {max}}&= p_{x_{\text {max}}}. \end{aligned}$$

Then the probability of inferring the wrong species tree with ASTRAL is bounded below by

$$\begin{aligned}&\ln P(\text {error})\\&\quad \ge \left[ - 2c' \ln c' - (1-2c') \ln (1-2c') + c' \ln p_{\text {max}}+ (1-c') \ln \frac{1-p_{\text {max}}}{2}\right] N' \\&\quad - \ln N' - \ln 4\pi c'(1-2c')^{1/2} - \frac{2-3c'}{12c'(1-2c')}\frac{1}{N'} + \frac{1}{12N'+1}, \end{aligned}$$

where $N' = |A_{x_{\text {max}}}||B_{x_{\text {max}}}||C_{x_{\text {max}}}||D_{x_{\text {max}}}|N$ and $c'$ is the nearest number to

$$\begin{aligned} c = \frac{1}{2+\sqrt{\frac{1-p_{\text {max}}}{2p_{\text {max}}}}}\end{aligned}$$

such that $c'N'$ is an integer.

It is also bounded above by

$$\begin{aligned} \ln P(\text {error})&\le \max _c \left\{ \left[ - 2c \ln c - (1-2c) \ln (1-2c) + c \ln p_{\text {min}}+ (1-c) \ln \frac{1-p_{\text {min}}}{2}\right] N \right. \\&\quad + \ln \frac{N+1}{3} + \ln \left( {\begin{array}{c}n\\ 4\end{array}}\right) - \ln 2\pi c(1-2c)^{1/2} \\&\quad \left. + \frac{1}{12N} - \frac{2}{12cN+1} + \frac{1}{12(1-2c)N+1} \right\} . \end{aligned}$$
